# CodonBERT: a BERT-based architecture tailored for codon optimization using the cross-attention mechanism

**DOI:** 10.1093/bioinformatics/btae330

**Published:** 2024-05-24

**Authors:** Zilin Ren, Lili Jiang, Yaxin Di, Dufei Zhang, Jianli Gong, Jianting Gong, Qiwei Jiang, Zhiguo Fu, Pingping Sun, Bo Zhou, Ming Ni

**Affiliations:** Changchun Veterinary Research Institute, Chinese Academy of Agricultural Sciences, State Key Laboratory of Pathogen and Biosecurity, Key Laboratory of Jilin Province for Zoonosis Prevention and Control, Changchun 130122, China; School of Information Science and Technology, Northeast Normal University, Changchun 130117, China; Changchun Veterinary Research Institute, Chinese Academy of Agricultural Sciences, State Key Laboratory of Pathogen and Biosecurity, Key Laboratory of Jilin Province for Zoonosis Prevention and Control, Changchun 130122, China; School of Information Science and Technology, Northeast Normal University, Changchun 130117, China; Changchun Veterinary Research Institute, Chinese Academy of Agricultural Sciences, State Key Laboratory of Pathogen and Biosecurity, Key Laboratory of Jilin Province for Zoonosis Prevention and Control, Changchun 130122, China; College of Veterinary Medicine, Northeast Agricultural University, Harbin 150038, China; Changchun Veterinary Research Institute, Chinese Academy of Agricultural Sciences, State Key Laboratory of Pathogen and Biosecurity, Key Laboratory of Jilin Province for Zoonosis Prevention and Control, Changchun 130122, China; School of Information Science and Technology, Northeast Normal University, Changchun 130117, China; School of Artificial Intelligence, Wuhan Technology and Business University, Wuhan 340000, China; Deartment of Regenerative Medicine, Institute of Health Service and Transfusion Medicine, Beijing 100850, China; Changchun Veterinary Research Institute, Chinese Academy of Agricultural Sciences, State Key Laboratory of Pathogen and Biosecurity, Key Laboratory of Jilin Province for Zoonosis Prevention and Control, Changchun 130122, China; School of Information Science and Technology, Northeast Normal University, Changchun 130117, China; School of Information Science and Technology, Northeast Normal University, Changchun 130117, China; Changchun Veterinary Research Institute, Chinese Academy of Agricultural Sciences, State Key Laboratory of Pathogen and Biosecurity, Key Laboratory of Jilin Province for Zoonosis Prevention and Control, Changchun 130122, China; Deartment of Regenerative Medicine, Institute of Health Service and Transfusion Medicine, Beijing 100850, China

## Abstract

**Motivation:**

Due to the varying delivery methods of mRNA vaccines, codon optimization plays a critical role in vaccine design to improve the stability and expression of proteins in specific tissues. Considering the many-to-one relationship between synonymous codons and amino acids, the number of mRNA sequences encoding the same amino acid sequence could be enormous. Finding stable and highly expressed mRNA sequences from the vast sequence space using in silico methods can generally be viewed as a path-search problem or a machine translation problem. However, current deep learning-based methods inspired by machine translation may have some limitations, such as recurrent neural networks, which have a weak ability to capture the long-term dependencies of codon preferences.

**Results:**

We develop a BERT-based architecture that uses the cross-attention mechanism for codon optimization. In CodonBERT, the codon sequence is randomly masked with each codon serving as a key and a value. In the meantime, the amino acid sequence is used as the query. CodonBERT was trained on high-expression transcripts from Human Protein Atlas mixed with different proportions of high codon adaptation index codon sequences. The result showed that CodonBERT can effectively capture the long-term dependencies between codons and amino acids, suggesting that it can be used as a customized training framework for specific optimization targets.

**Availability and implementation:**

CodonBERT is freely available on https://github.com/FPPGroup/CodonBERT.

## 1 Introduction

Codon optimization is a crucial aspect of vaccine and protein design (Boël *et al.* 2016), especially in the field of mRNA vaccines. This importance stems from the inherent challenges associated with codon usage, where rational codon selection can enhance stability and protein expression (Hanson and Coller 2018). In particular, codon preference may vary among different organisms and tissues (Ikemura 1985), which imposes new requirements on codon optimization in terms of the ability to extract or learn organism- or species-specific “dialects”.

Several codon optimization tools based on different strategies have been developed in the past. Commonly used tools such as JCAT (Grote *et al.* 2005) and Optimizer (Puigbò *et al.* 2007), are based on the “one amino acid—one codon” strategy. These methods select the most frequently used codon in the host for each amino acid according to the codon usage table. Zhang *et al.* (2023) proposed LinearDesign, which integrates stability and expression optimization using lattice parsing, a classic natural language processing technology. Jain *et al.* (2022) and Fu *et al.* (2020) treated the codon optimization problem as a machine translation problem using supervised deep learning models. Recently, Hernandez-Alias et al. proposed CUSTOM and demonstrated the existence of codon preferences in tissue-specific protein synthesis (Hernandez-Alias *et al.* 2023).

However, optimizing codons for specific organisms and tissues is a complex task that is influenced by factors such as translation elongation efficiency, translation accuracy, and translation initiation signals (Xia 2021). It is extremely challenging for codon optimization to achieve optimal performance in multiple optimization targets. With the widespread use of transcriptomic high-throughput sequencing, it may be an interesting idea to learn the characteristics of “dialects” directly from naturally highly expressed codon sequences.

Here, we present CodonBERT, which uses the cross-attention mechanism to capture the contextual relationship between codons and amino acids. The model was trained using high-level transcripts per kilobase per million mapped reads (TPM) RNA-seq data from the Human Protein Atlas (HPA) (Sjöstedt *et al.* 2020). The study demonstrates that CodonBERT performs well in terms of accuracy and the ability to learn codon sequence characteristics. The architecture is flexible and can be trained on specific data for organism- or species-specific codon optimization tools.

## 2 Materials and methods

**Figure 1.**
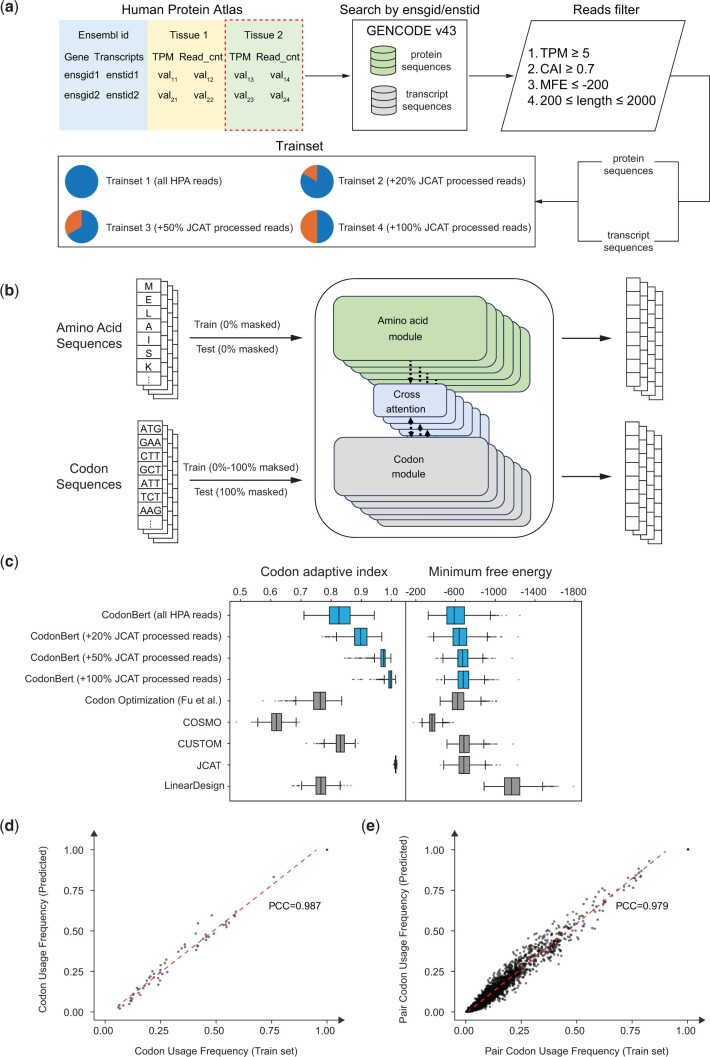
The overview of this study. (a) The data processing workflow on the HPA dataset. (b) The architecture of CodonBERT. (c) The performance of codon adaptation index (CAI) and minimum free energy (MFE). The Performance of CAI and MFE was estimated on each optimized read in the test set using EMBOSS and RNAfold, respectively. (d) The correlation between the frequency of codon usage in the predicted sequences (*y*-axis) in the test set and the frequency in the training data (*x*-axis). (e) The correlation between the frequency of pair codon usage in the predicted sequences (*y*-axis) in the test set and the frequency in the training data (*x*-axis).

We downloaded transcript expression levels and Ensembl identifiers for 186 human tissue samples based on RNA-seq data from the HPA (www.proteinatlas.org). Protein and transcript sequences were then extracted from the GENCODE database (Frankish *et al.* 2021) using the gene identifier and transcript identifier. We filtered the transcripts with TPM values beyond 5 (top 5%, Supplementary Fig. S1). Then, the codon adaptation index (CAI) and minimum free energy (MFE) for each sequence were calculated using EMBOSS v6.6.0 (Olson 2002) and ViennaRNA v2.6.4 (Lorenz *et al.* 2011) for filtering purposes. Finally, four training datasets were then constructed with different proportions of JCAT-optimized sequences. The detail of data preprocessing is described in Fig. 1a and Supplementary material.

The architecture of CodonBERT is based on ProteinBERT (Brandes *et al.* 2022), with some modifications to accommodate the contextual relationships for codon sequences (Fig. 1b and Supplementary Fig. S2). The two main modifications are as follows: (1) the right-side network was rebuilt to match the encoder on the left side; (2) codon tokens are now used as both keys and values in the cross-attention mechanism, while the protein sequence serves as the query. During the training process, CodonBERT learns codon usage preferences and contextual combination preferences through randomly masked codon and amino acid tokens.

To predict codon sequences only from protein sequences, we implemented a training strategy that involved stepwise masking of the entire codon sequences in a batch. Initially, during the first 15 training epochs, all codon sequences were fed into the network. Then, every 15 epochs, 5% of the codon sequences in each batch were replaced with a tensor of full zeros. Finally, when the training epoch exceeded 300, all codon sequences were replaced with zeros.

## 3 Results

The performance of CodonBERT was assessed in terms of accuracy, CAI, and MFE. Trained separately on four datasets with different proportions of JCAT-optimized sequences, CodonBERT consistently achieved accuracies with a median of 100% (Supplementary Fig. S3, Supplementary Table S1). This performance is superior to that of the recurrent neural network-based model (training accuracy and test accuracy of BiLSTM-CRF-a were 76% and 52%, respectively, as reported by Fu *et al.* 2020). Figure 1c shows that JCAT uses the most prevalent codon in highly expressed genes, resulting in a CAI of 1. CodonBERT’s CAI increased as the proportion of JCAT-optimized sequences increased, indicating its ability to capture sequence characteristics. CodonBERT performed comparably to other tools in terms of MFE, except LinearDesign, which focuses on stability improvement.

To further investigate whether CodonBERT can capture sequence characteristics from the training set, we calculated the Pearson’s correlation coefficients (PCCs) of codon and pair codon usage between the training set and the test set. We also summarized the codon usage and pair codon usage for the respective training sets used in CUSTOM and Fu et al.’s study. We then used these tools to predict codon sequences on the same test set, where the codon usage was recorded. As a result, CodonBERT achieved PCCs of 0.987 and 0.979 (Fig. 1d), outperforming CUSTOM (0.870 and 0.806) and Fu et al.’s Codon Optimization (0.804 and 0.671, Supplementary Fig. S4). The result showed that CodonBERT can be used as a customized tool to achieve specific optimization goals by training on in-house data.

## 4 Conclusion

In this pilot study, we propose a deep learning method for codon optimization, called CodonBERT. The results showed that CodonBERT can efficiently learn codon usage preferences from the training data. Therefore, CodonBERT is a versatile model that can be flexibly trained on specific data for specific purposes. However, there are some limitations to consider. The HPA database provides RNA-seq data from various tissues, allowing the development of multiple models for tissue-specific codon optimization. However, we have found that using TPM to filter tissue-specific data is not optimal. The codon preferences among different tissues are too similar (see Supplementary Table S2). In addition, we only evaluated the model performance with two common metrics, CAI and MFE, without performing any wet experiments. Furthermore, we did not provide a graphical user interface for the application, which could be further developed. In conclusion, with the development of a wide variety of mRNA vaccines, CodonBERT may be a promising method for custom codon optimization.

## Supplementary Material

btae330_Supplementary_Data
