# Supplementary material for: CodonBERT: a BERT-based architecture tailored for codon optimization using the cross-attention mechanism
Source: Bioinformatics. 2024 May 24;40(7):btae330. doi: 10.1093/bioinformatics/btae330 (PMC11226863; doi:10.1093/bioinformatics/btae330)
Supplement: btae330_Supplementary_Data [file btae330_supplementary_data.docx]

# Supplementary Information to:

CodonBERT: a BERT-based architecture tailored for codon optimization using the cross-attention mechanism

## Supplementary Methods

1. **Data collection**

The *transcript_rna_tissue.tsv* file was retrieved from the Human Protein Atlas (version 23.0, accessible at https://www.proteinatlas.org, Sjöstedt *et al.*, 2020). In this tab-separated file, each row represents a transcript and includes essential information such as the Ensembl gene identifier ("ensgid"), Ensembl transcript identifier ("enstid"), transcript per million ("TPM"), and estimated counts ("est_counts") specific to the analyzed tissue-level sample ("sample_name.sample_id"). Notably, the identifiers used are sourced from Ensembl version 109. Subsequently, leveraging these identifiers, we gathered a dataset comprising 40,489 protein sequences and their corresponding transcript sequences from the file *gencode.v43.pc_translations.fa.gz* (GENCODE Human Release 43, accessible at www.gencodegenes.org, Frankish *et al.*, 2021). This dataset serves as a foundational resource for our analyses, aligning with the latest genomic annotations and providing a comprehensive representation of protein and transcript relationships.

1. **Data processing**

We first summarized the distribution of transcript per million (TPM) levels for the collected sequences, as shown in Supplementary Figure 1A. The top 5% of sequences exhibited TPM values beyond 5. Thus, a threshold of 5 was applied to obtain the highly expressed sequences, ensuring a focused selection based on elevated expression levels. The sequences obtained from GENCODE were in FASTA format, where each sequence’s position information was recorded. With this information, we systematically extracted protein-coding sequences. To ensure the integrity of protein sequences, we translated the coding sequences into protein sequences using Biopython v1.8.1 (Cock *et al.*, 2009) and then ascertained the identity between translated sequences and sequences from the file *gencode.v43.pc_translations.fa.gz* by direct comparison.

To construct the training set, a series of processing steps were executed on the collected data, encompassing length filtering, minimum free energy (MFE) and codon adaptation index (CAI) filtering, and the incorporation of sequences optimized via JCAT. First, we reserved amino acid sequences ranging in length from 200 aa to 2000 aa. This range was chosen for two reasons. Currently, the input sequence length demanded by larger BERT-type models typically does not exceed 2048. This constraint is primarily dictated by the training speed of the self-attention mechanism (Ding *et al.*, 2020). Excessively long sequences can significantly impede training efficiency. Simultaneously, to enable comprehensive learning of sequence features, sequences that are too short (with lengths less than 200 aa) are also excluded. Second, we removed sequences with low CAI and high MFE values. CAI and MFE were calculated using EMBOSS v6.6.0 (Olson, 2002) and ViennaRNA v2.6.4 (Lorenz *et al.*, 2011). The distribution of codon adaptation index (CAI) and minimum free energy (MFE) for all sequences was illustrated in Supplementary Figure 1B. The CAI values for the majority of sequences were primarily concentrated within the range of 0.7 to 0.85. Additionally, the MFE values predominantly fell within the range of -400 to -100. As a result, only sequences meeting the criteria of CAI ≥ 0.7 and MFE ≤ -200 were retained as training data for the model. This stringent selection process ensures that only sequences with favorable codon adaptation indices and sufficiently stable secondary structures are utilized for training, thereby enhancing the robustness and efficacy of the model. Four training sets were constructed from the screened codon sequences and those optimized by JCAT with four different mixing ratios of 1:0, 1:0.2, 1:0.5, and 1:1. The number of codon sequences in the four training sets was 10k, 12k, 15k, and 20k, respectively. The test set contains 1309 amino acid sequences between 500 and 600AA in length, and none of these amino acid sequences are identical to the amino acid sequences corresponding to the codon sequences of the training set.

Due to the various length, amino acid and codon sequences were padded into the same length. We used 24 tokens to encode amino acid sequences, including padding (0), start (1), end (2), and 20 amino acids (ranging from 3 to 22). Besides, we use ‘X’ (23) to correspond to the stop codon. As for codon sequences, we used 67 tokens, including padding (0), start (1), end (2), and 64 different codons (ranging from 3 to 66). For each random mask position in the sequence, we generated a tensor with the same shape as the sequence, wherein a value of 1 indicates the presence of the mask, while a value of 0 signifies no mask.

1. **Model framework**

CodonBERT is inspired by ProteinBERT (Brandes *et al.*, 2022). CodonBERT is composed of six blocks, each featuring two inputs and two outputs. The left input corresponds to the embedding of amino acid sequences, while the right input represents the embedding of codon sequences (see Supplementary Figure 2). Every block comprises two parallel sub-networks, and the embeddings of amino acid sequences undergo processing through wide convolution, narrow convolution, and self-attention layers for feature extraction. These three outcomes are concatenated with the results of the right sub-network's feature extraction of codon sequence embedding information, achieved via linear layers and the GELU activation function. Subsequently, the concatenated output undergoes normalization through a dedicated layer. The normalized result then proceeds through linear layers, the GELU activation function, and layer normalization, serving as the input for the left sub-network in the subsequent block. In detail, both the wide and narrow CNN kernel sizes were set to 9, with a wide convolution stride of 5 and the default value of 1 for the narrow convolution stride. The channels were set to 512 to effectively capture features during training.

Concurrently, the right sub-network takes as input the outcome of the interaction between amino acid and codon sequences. The output of the last layer from the left sub-network in each block acts as the Query input for cross-attention, while the codon sequence embedding functions as the Key and Value inputs. This configuration enables the amino acid sequence to extract information from the codon sequence, facilitating amino acid-to-codon prediction functionality. The right sub-network further refines the cross-attention output through wide convolution and narrow convolution operations, combining the results and subjecting them to normalization. The normalized output is then processed through linear layers, the GELU activation function, and layer normalization, serving as the input for the right sub-network in the subsequent block. Following the completion of six blocks, the outputs of the left and right sub-networks undergo separate processing through linear layers to facilitate classification prediction.

Initially, random position-based masking was implemented at the codon level, wherein each codon underwent masking individually, with a designated masking ratio of 5%. This method involves randomly concealing information at specific positions along the codon sequence. Subsequently, a sequence-level mask is applied. A predetermined proportion of sequences in each batch were entirely covered with tensors consisting of zeros. This proportion gradually increases with training epochs. Specifically, the mask ratio elevates by 5% every 15 epochs. This iterative process is designed to ensure that the model can effectively predict the codon sequence solely from the amino acid sequence. By gradually increasing the mask ratio, the model is incentivized to acquire resilient representations that capture crucial sequence patterns even when certain parts of the input are concealed. This improves its ability to generalize.

We incorporate the cross-attention mechanism into the network architecture, enabling the model to effectively leverage the interaction between protein sequence information and mRNA sequence information. This attention mechanism, widely applied and validated in Natural Language Processing (NLP) tasks, aids the model in identifying and focusing on pertinent information while disregarding irrelevant data, thereby enhancing its performance and generalization capability. The attention mechanism relies on three key parameters: Q (Query), K (Key), and V (Value). By computing the similarity between Q and K, the model obtains an attention weight matrix, which is then utilized to weight and aggregate V, resulting in the final output. In our task, we utilize protein sequence information as Q and the corresponding high-expression mRNA sequence information as K and V. This configuration enables the model to effectively align and extract relevant information from both protein and mRNA sequences, enhancing its ability to make accurate predictions.

The CodonBERT model was trained with specific parameter configurations, including a batch size of 32 and a learning rate of 3e-4. Early stopping, with a patience of 10 epochs, was implemented to mitigate overfitting and enhance model performance. Optimization during training was facilitated using the Adam optimizer and Cross Entropy Loss function. The training process utilized a 1080TI GPU and spanned a total of 320 epochs. This comprehensive training regimen required approximately 220 hours to complete, ensuring thorough optimization and convergence of the model.

1. **Tissue-specific analysis**

In this experiment, we aimed to train tissue-specific models by creating tissue-specific training datasets. We selected three datasets from the original dataset that contained transcripts per million (TPM) information. These datasets included TPM values greater than 5 in kidney, lung, and bone tissues, respectively, and were used as tissue-specific training data. Using the same model framework, three training sets were utilized to train separate model parameters for achieving high TPM codon optimization in kidney, lung, and bone tissues. However, we found that the Pearson correlation coefficients of codon usage and codon pair usage between any two tissues were higher than 0.99 (Supplementary Table 2). This suggests that reliable data is needed for further tissue-specific codon optimization models.

## Supplementary Figures


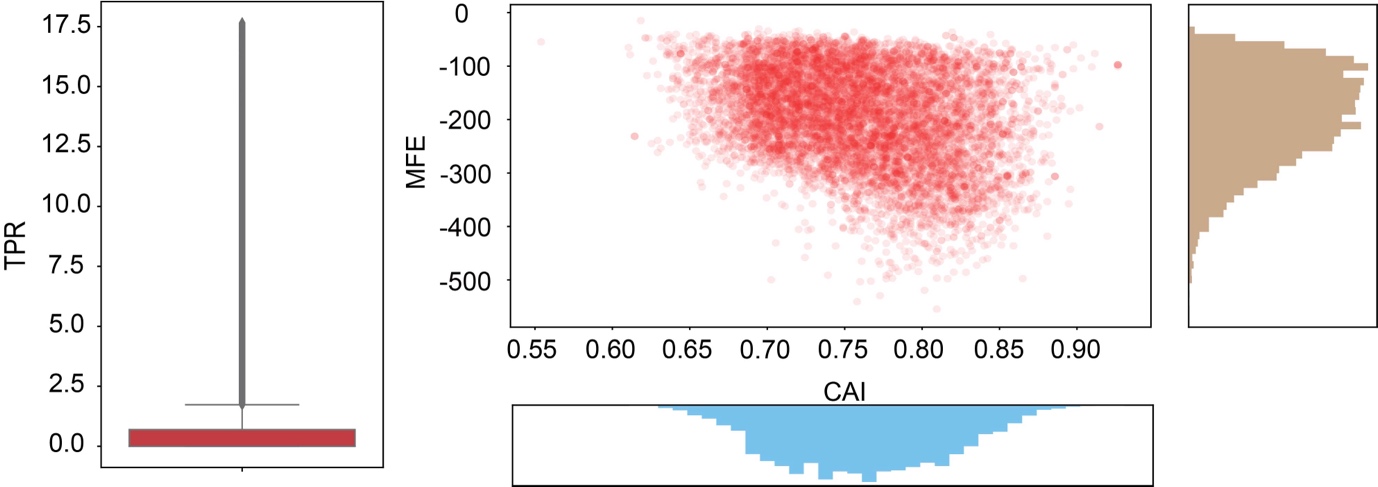


**Supplementary Figure 1**. The distribution of transcript per million (TPM), minimum free energy (MFE), and codon adaptation index (CAI) in the collected data.


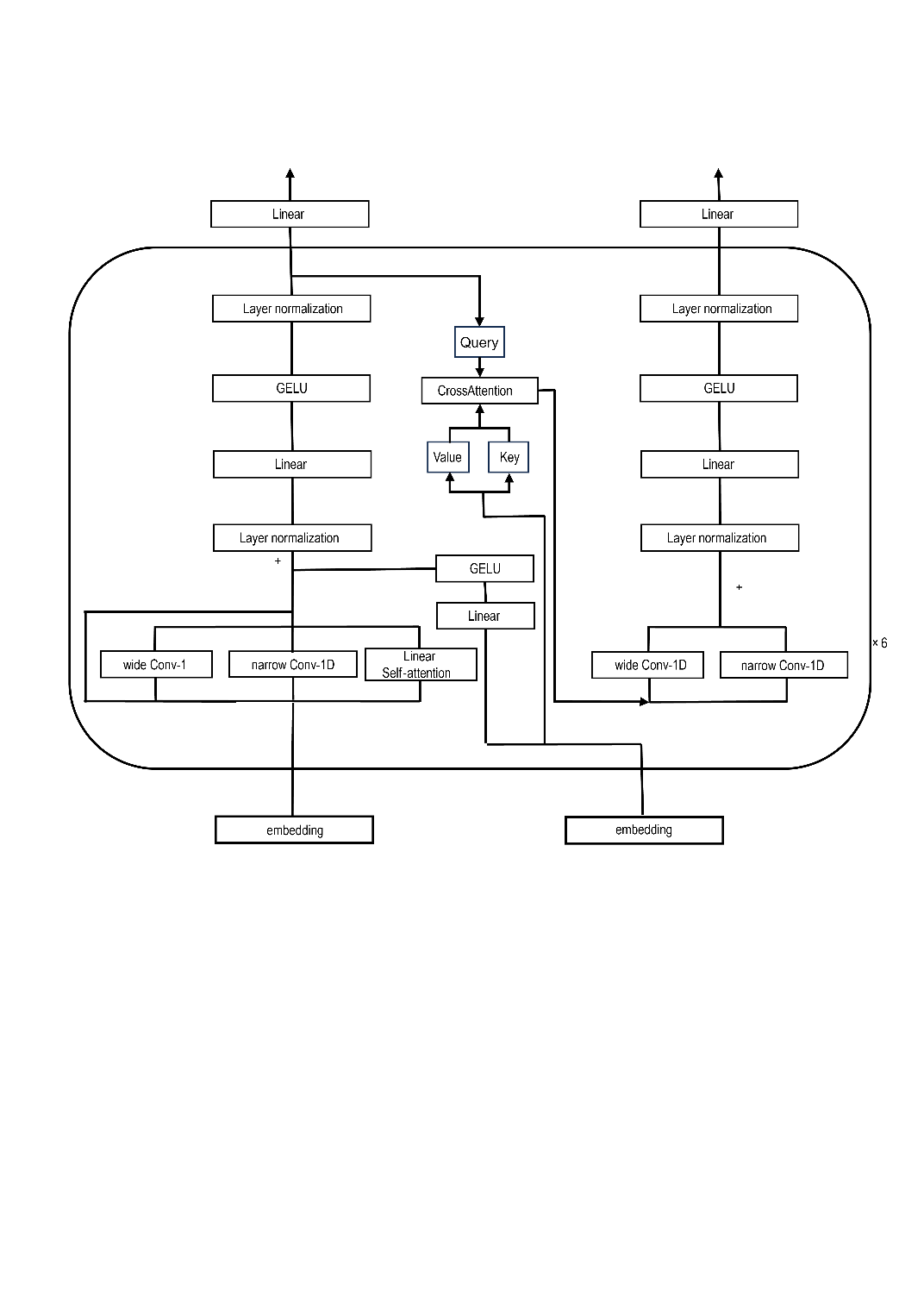


**Supplementary Figure 2**. The architecture of CodonBERT. CodonBERT is composed of six blocks, each featuring two inputs and two outputs. The left input corresponds to the embedding of amino acid sequences, while the right input represents the embedding of codon sequences.


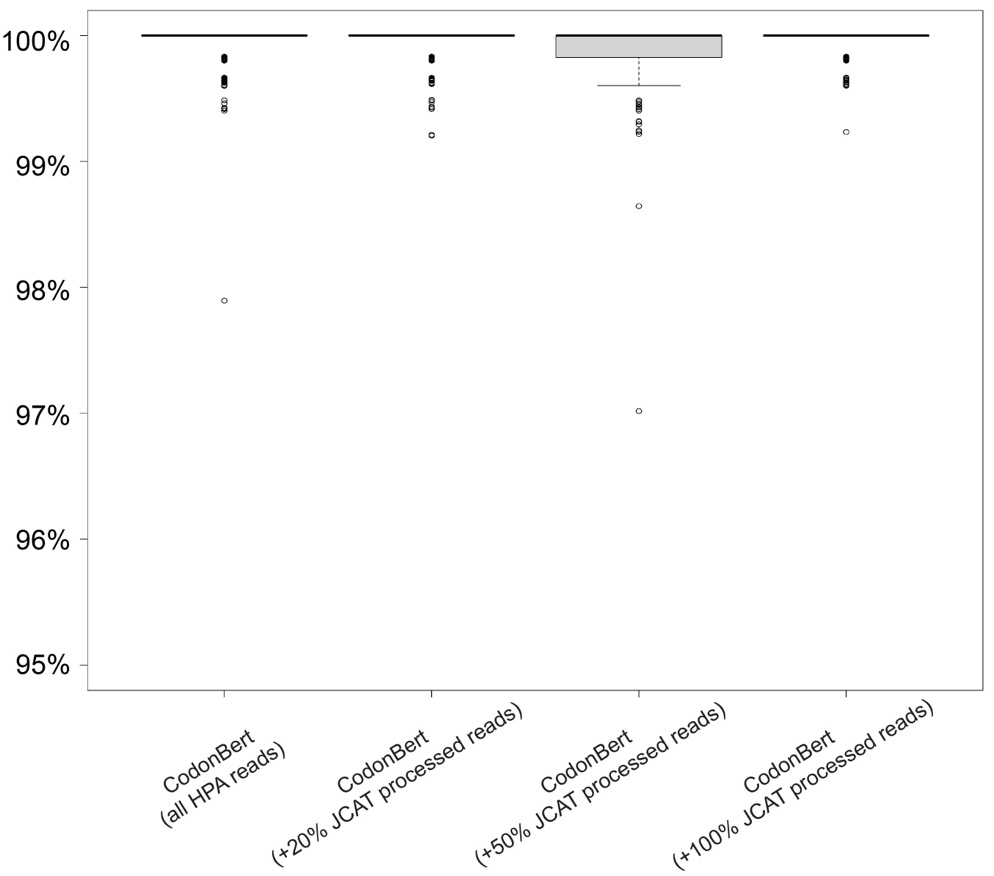


**Supplementary Figure 3**. The accuracy performance of CodonBERT. We trained four models with various training sets (x-axis). The accuracy is calculated as 1 minus the proportion of non-synonymous codon predictions in the predicted sequences.


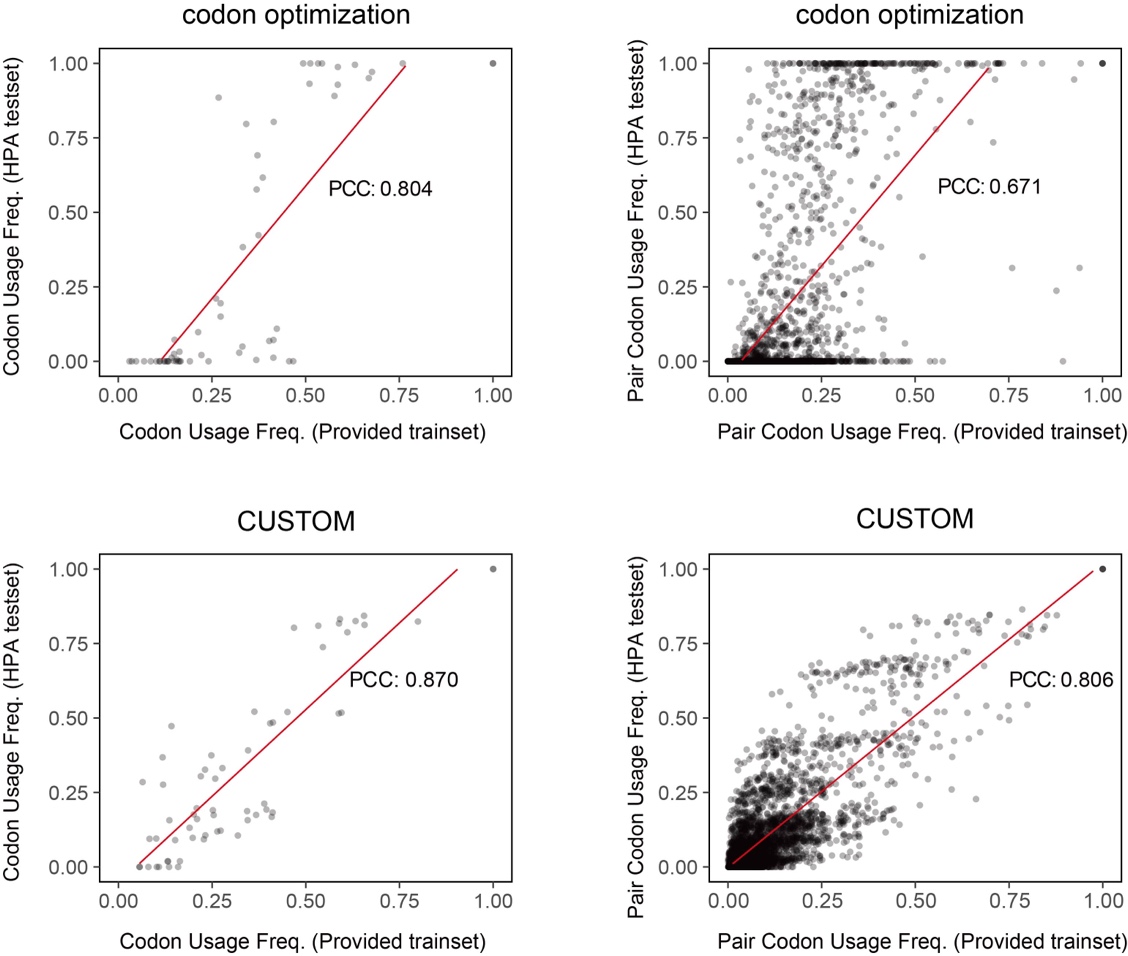


**Supplementary Figure 4.** The scatter plots show codon and pair codon usage frequencies of sequences from provided training sets and predicted sequences using the codon optimization (Fu *et al.*, 2020) and CUSTOM (Hernandez-Alias *et al.*, 2023). For CUSTOM, we used the gene identifiers provided in the supplementary material to download the sequence and summarize the frequencies of codons and codon pairs.

## Supplementary Tables

**Supplementary Table 1.** The accuracy of predicted amino acid sequences using CodonBERT on the test set.

|  | 10,000 HPA reads | +20% JCAT-optimized reads | +50% JCAT-optimized reads | +100% JCAT-optimized reads |
| --- | --- | --- | --- | --- |
| count | 1309 | 1309 | 1309 | 1309 |
| mean | 99.96% | 99.97% | 99.93% | 99.97% |
| std | 0.0011 | 0.0009 | 0.0014 | 0.0008 |
| min | 97.89% | 0.992048 | 97.02% | 0.992337 |
| 25% | 100% | 100% | 99.83% | 100% |
| 50% | 100% | 100% | 100% | 100% |
| 75% | 100% | 100% | 100% | 100% |
| max | 100% | 100% | 100% | 100% |

**Supplementary Table 2.** The Pearson’s Correlation Coefficients (PCCs) of codon and pair codon usage of high-TPM sequences from different tissues.

|  | PCC of codon usage Frequency | PCC of pair codon usage Frequency |
| --- | --- | --- |
| kidney-lung | 0.9984 | 0.9981 |
| kidney-skeletal | 0.9974 | 0.9966 |
| lung-skeletal | 0.9998 | 0.9992 |

## References

Brandes,N. *et al.* (2022) ProteinBERT: a universal deep-learning model of protein sequence and function. *Bioinformatics*, **38**, 2102–2110.

Cock,P.J.A. *et al.* (2009) Biopython: freely available Python tools for computational molecular biology and bioinformatics. *Bioinformatics*, **25**, 1422–1423.

Ding,M. *et al.* (2020) CogLTX: Applying BERT to Long Texts. In, *Advances in Neural Information Processing Systems*. Curran Associates, Inc., pp. 12792–12804.

Frankish,A. *et al.* (2021) GENCODE 2021. *Nucleic Acids Research*, **49**, D916–D923.

Fu,H. *et al.* (2020) Codon optimization with deep learning to enhance protein expression. *Sci Rep*, **10**, 17617.

Hernandez-Alias,X. *et al.* (2023) Using protein-per-mRNA differences among human tissues in codon optimization. *Genome Biology*, **24**, 34.

Sjöstedt,E. *et al.* (2020) An atlas of the protein-coding genes in the human, pig, and mouse brain. *Science*, **367**, eaay5947.
